# Supplementary figures and images for: The nuclear basket nucleoporin MLP1 is required to maintain nuclear integrity, and mitotic fidelity in Trypanosoma brucei
Source: PLoS Negl Trop Dis. 2026 Jun 22;20(6):e0013922. doi: 10.1371/journal.pntd.0013922 (PMC13298991; doi:10.1371/journal.pntd.0013922)

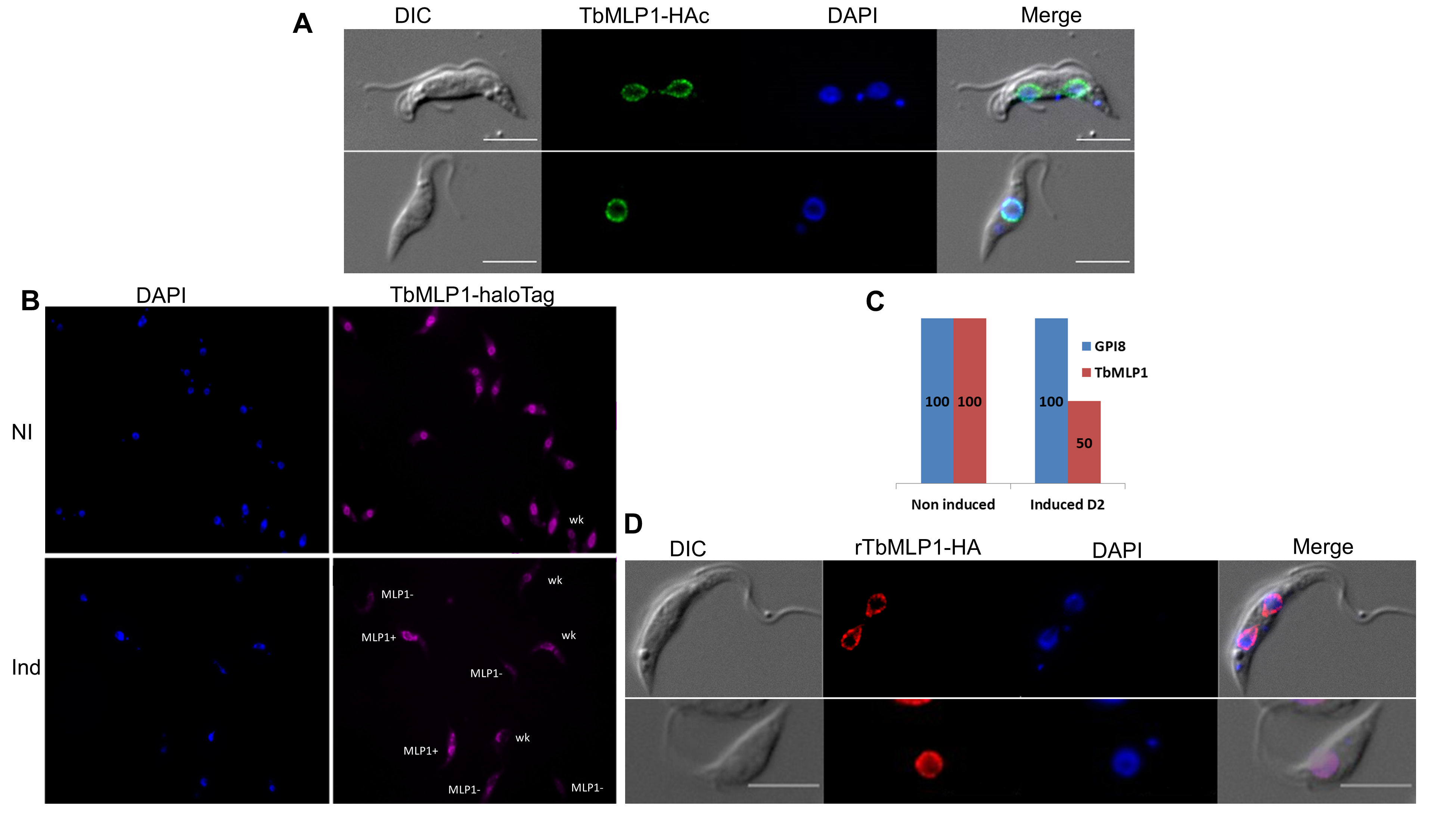

Supplement: S1 Fig — (A) TbMLP1-HAc localization in T. brucei PCF using and anti-HA antibody (Green). DAPI was used to visualize DNA (blue). Mitotic cells (upper lane), interphasic cells (lower lane). Bar: 5 mm. Fluorescence was visualized using a Zeiss Z2 microscope and acquired as series of Z-axes. (B) TbMLP1-HaloTag labeling in non-induced RNAi parasites (NI) and two days post tetracycline induction (Ind). The signal was detected using an immunofluorescence assay. TbMLP1-HaloTag labeling was classified into three categories; positive signal (MLP1+), weak signal (wk) and negative signal (MLP1-). In these representative fields, all non induced NI cells are MLP1 + , except for one cell displaying a weak signal. After 2 days of induction, all three categories become visible; with the vast majority of cells (88%) exihibiting either a negative or weak MLP1 signal and 12% remaining MLP1 positive. (C) qRTPCR results performed on cDNAs prepared from TbMLP1 RNAi cell line either non-induced (NI) or induced two days with tetracycline (ind), using primers specific to TbMLP1 and, as a control, primers specific to the housekeeping gene GPI8. (D) TbMLP1-HA recodonized version (rTbMLP1-HA) localization in TbMLP1 RNAi cell line visualized with an anti-HA antibody (Red). DAPI was used to visualize DNA (blue). interphasic cells (upper lane), mitotic cells (lower lane). (TIF) [file pntd.0013922.s001.tif]

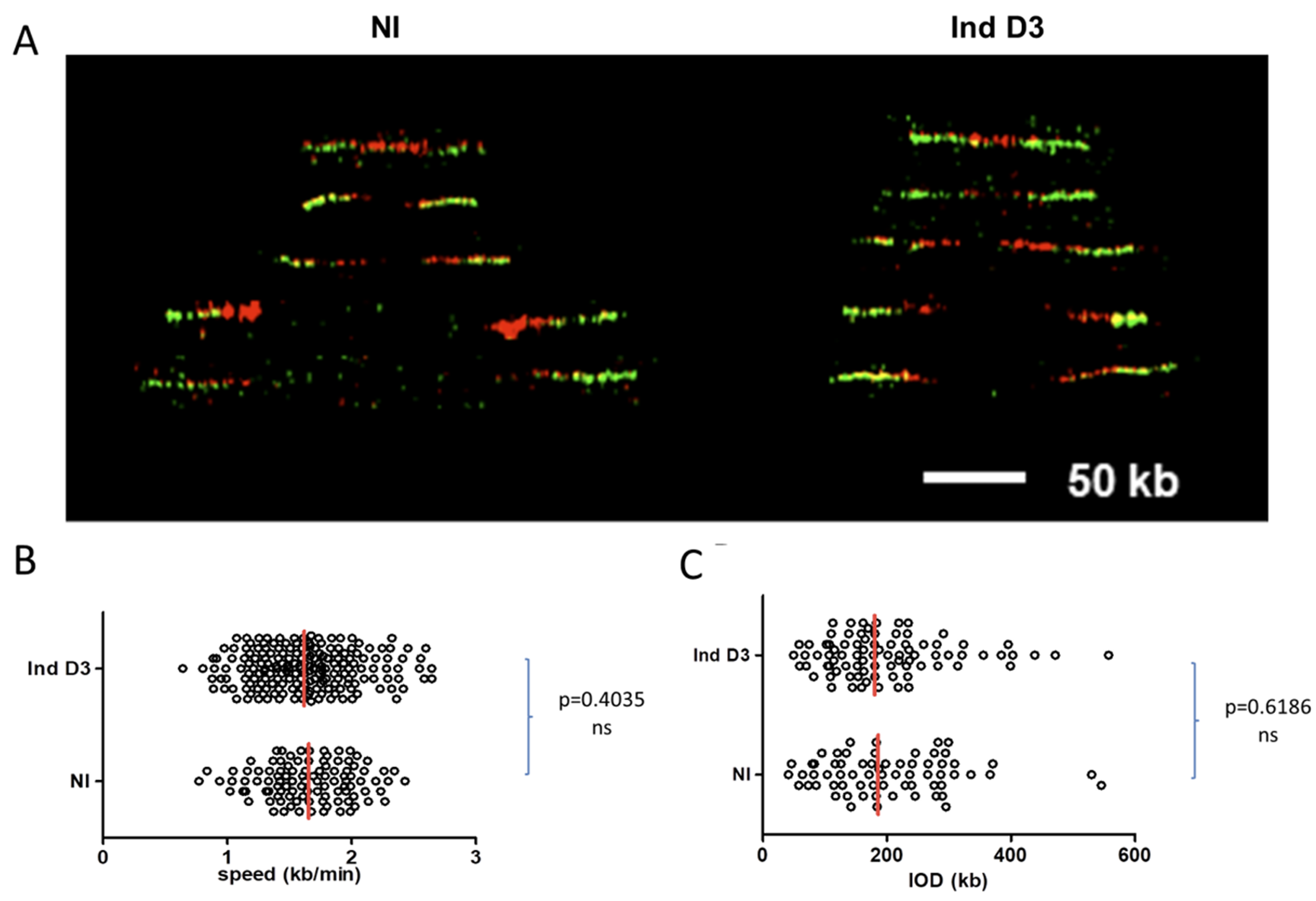

Supplement: S2 Fig — (A) Representative bidirectional replication forks from TbMLP1 RNAi non-induced (NI) or tetracycline induced (Ind D3) taken from different microscopic fields, artificially assembled and centred on the position of the presumed origins. Red tracks: IdU, green tracks: CldU. Scale bar: 50 kb. (B) Comparative analysis of the velocity of replication forks in TbMLP1 RNAi non-induced (NI) or tetracycline induced (Ind D3). (C) Comparative analysis of the IOD in TbMLP1 RNAi non-induced (NI) or tetracycline induced (Ind D3). p values were calculated using two-tailed Mann-Whitney test (p < 0.05 was taken as significant). (TIF) [file pntd.0013922.s002.tif]
